# Nursing students’ development of clinical competence through the use of reflection prompts based on the nursing process during clinical education: a qualitative longitudinal study

**DOI:** 10.1186/s12909-026-08989-z

**Published:** 2026-04-01

**Authors:** Ulrika Löfgren, Britt-Marie Wälivaara, Ulrica Strömbäck, Birgitta Lindberg

**Affiliations:** Institution of Health, Education and Technology, Division of Nursing and Radiography, Luleå university of technology, Luleå, 97187 Sweden

**Keywords:** Clinical competence, Clinical education, Learning, Nursing process, Reflection, Prompts, Qualitative

## Abstract

**Aim:**

The aim of the study was to identify patterns in nursing students’ development of clinical competence through the use of reflection prompts based on the nursing process during their clinical education.

**Design:**

This study’s design was a qualitative longitudinal small-scale intervention study with an inductive approach.

**Methods:**

Data were collected between November 2024 and January 2025 through 18 semi-structured individual interviews with six nursing students in their sixth semester during their final clinical placement. Each student was interviewed three times during their clinical education: before, during, and after using a card with reflection prompts. The data was analysed using a pattern-oriented analysis.

**Results:**

The analysis resulted in four individual and shared patterns illustrating students’ development of clinical competence over time through the use of reflection prompts during their clinical education. The main patterns revealed that the reflection prompts enhanced understanding of the nursing process as an integrated part of clinical practice, developed independent reflective thinking in the professional role, and advanced a fundamental structure for nursing practice. Nevertheless, continual reflective supervision is an important prerequisite for optimal development.

**Conclusion:**

Reflection prompts based on the nursing process show a potential to support nursing students’ learning and development of clinical competence during their clinical education. Further research is needed to refine and strengthen the model and to explore how it can be implemented in nursing education.

## Background

In recent years, the number of weeks of clinical placement in Swedish nursing education programmes has significantly increased in response to European Union (EU) directives [1, 2]. This expansion provides students with more extensive hands-on experience, which increases their ability to apply theoretical knowledge in real care settings; however, it also presents challenges for both universities and healthcare providers related to ensuring quality and continuity in learning. As a result, nursing education must develop didactic learning strategies which support students in developing their competence during clinical education while also equipping clinical supervisors with the tools to effectively guide and facilitate this learning process.

Developing a sufficient level of clinical competence in the final year of nursing education is important for the transition from nursing student to registered nurse [3]. The development of clinical competence is not a straightforward process, rather, it is an iterative journey which evolves over time through continual practice, repetition, and experience [4]. The findings from one longitudinal study showed that students’ understanding of the complexity of nursing develops gradually, with deeper knowledge and a more holistic perspective in the later stages of education [5]. Student-centred clinical education which supports reflection and the integration of theory and practice enhances learning and professional growth [6]. In particular, reflecting on core concepts in nursing increases students’ understanding and supports their professional development, as it helps them articulate and deepen their knowledge [7]. Reflective, critical thinking helps students develop a strong professional identity for their future role as nurses [8] and improve their patient safety competencies [9].

The nursing process is a theoretical problem-solving model used by nurses to systematically assess, plan, and provide safe and effective care for patients and their families [10]. According to the competency description of the Swedish Higher Education Ordinance [11] and the Swedish Society of Nursing [12], upon graduation, students should be able to independently apply the nursing process in clinical practice. In Sweden, the nursing process is incorporated into clinical education assessment tools [13] and in learning outcomes for the nursing degree. However, previous studies have indicated that both students and supervisors often struggle to apply the nursing process in practice, and it is seldom actively reflected upon [14, 15]. Nevertheless, when students grasp its meaning, it often serves as an eye-opener which deepens their understanding of nursing [16]. Students have described that reflection deepens their understanding of caring theory and nursing practice, thereby helping them integrate personal values with professional competence [17].

Didactic models combining reflexive and critical approaches to nursing are the foundation of nursing students’ learning in clinical education [18, 19]. A review by Berndtsson et al. [20] found that work-integrated learning supports the integration of theory and practice by enhancing students’ critical thinking and stimulating reflection. Supportive didactic tools in clinical education which encourage conscious, structured reflection can promote both learning and caring processes [21–23]. In addition, students’ and supervisors’ active engagement in clinical processes improves the former’s critical reasoning, decision-making, and overall competence [24]. Teaching strategies which connect the nursing process and critical thinking can support the student’s synthesis of theoretical knowledge and clinical competencies [25]. This calls for collaboration between universities and clinical settings to develop shared didactic strategies [5, 21]. To this end, this study aims to explore whether reflection prompts grounded in the nursing process can help students link theory and practice by encouraging structured reflection and dialogue and thereby deepen students’ understanding, support their professional growth, and foster the development of clinical competence for lifelong learning.

### Aim

The aim of the study was to identify patterns in nursing students’ development of clinical competence through the use of reflection prompts based on the nursing process during their clinical education.

## Methods

### Study design

This intervention study was conducted as a short-term and small-scale qualitative longitudinal study (QLS) employing an inductive approach. A QLS is particularly well-suited for repeated data collection within the same sample of participants, as it enables a deeper understanding of change processes both at the individual level and within the group [26]. Exploring experiences over time for individuals and across groups provides a holistic and in-depth understanding which goes beyond what can be captured at a single point in time [27]. It also enables researchers to explore both short- and longer-term processes of change, which are often missed in cross-sectional designs [28]. In the current context, this approach can provide a nuanced understanding of how and why the intervention may influence students’ development. QLS is well-suited for evaluating intervention outcomes [28, 29].

With a conceptualisation of change as “from-through,” the process of transformation can be captured [30]. In this study, time is viewed as fluid, and a particular aspect is followed over time to understand the changes which occur, as it is only through the passage of time that change can be truly understood [26]. In this study, time is understood as both objective and subjective. Each situation is seen both as a point on a predefined timeline and as a personal experience shaped by the individual’s own interpretations, past experiences, and expectations [30].

### The intervention

**Fig. 1:**
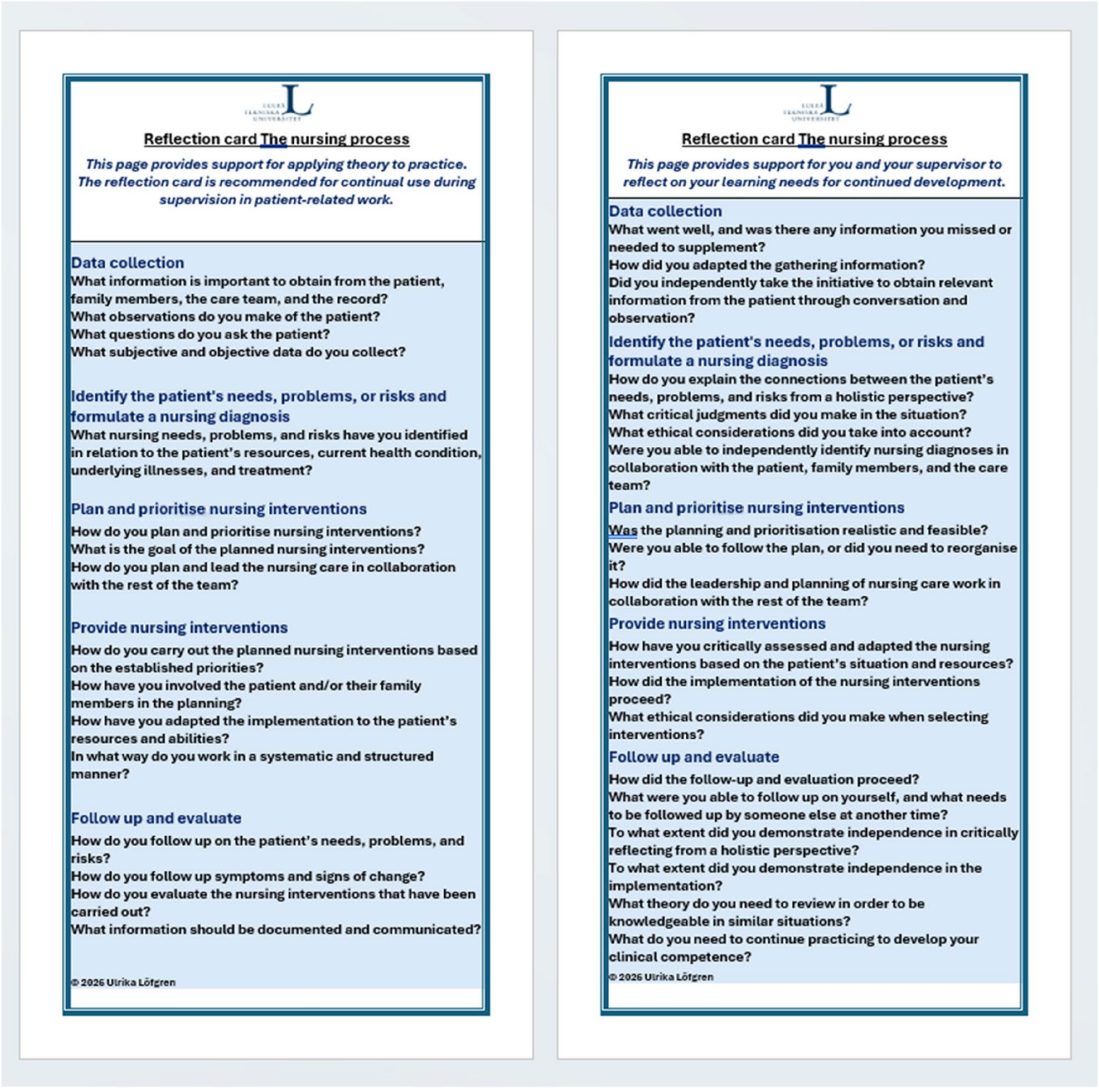
The card with reflection prompts

Theoretically grounded in the nursing process, the intervention consisted of targeted prompts aligned with the key components of the nursing process, these prompts were designed to support structured reflection. The content in the prompts is informed by findings from previous studies conducted by the author team [14–16]. The reflection prompts were distributed to all potential students in the final course in the form of a pocket-sized double-sided card. One side of the card featured questions based on the nursing process to support theory–practice integration and the other side of the card included prompts for self-reflection on personal learning and development (Fig. 1). Students were encouraged to use the card daily during patient care.

### Participants and setting

The study included six nursing students from a university in northern Sweden who were completing their final clinical placement before graduation. Voluntary students were recruited through purposive sampling from the final course with 45 eligible students in the nursing programme, which included a six-week clinical placement. Participants included five women and one man, with a median age of 41 years (range 24–47 years). They completed their clinical placements in medical and surgical wards across five hospitals. Like all final-year nursing students, each student was supervised by one to two clinical supervisors and took mid-term, practical, and final examinations with a clinical teacher. The learning objectives were based on the nursing process and aligned with national degree requirements for registered nurses, as outlined in the Swedish Higher Education Act and the Swedish Higher Education Ordinance [11, 31].

### Procedure

Approval from the head of nursing education was obtained before students were addressed. All 45 students in the final course received study and intervention details via video link, supplemented by written information in their learning platform, at the start of the course. This was restated in person by the first and last authors, who also distributed two reflection cards for all students in the course to use voluntary, one for personal use, and one for their supervisor. An informational manual accompanied the card. Participation in the study was also voluntary, and interested students contacted the researchers and provided their written consent. Clinical teachers were informed about the voluntary intervention in a digital meeting and were asked to share the information with supervisors. A digital version of the reflection card and the manual was published on the clinical education website.

### Data collection

Data was collected via semi-structured audio-recorded video interviews between November 2024 and January 2025 by the first author. Each student took part in three interviews—one before, one during, and one after their six-week clinical placement, for a total of 18 interviews. Interviews ranged from 10 to 28 min with a mean of 15 min, and they were transcribed verbatim. The first interview served as a baseline for capturing changes over time. The interview questions (Table 1) encouraged elaboration through continual follow-up prompts.

**Table 1:** Sample questions from the interview guides

Interview 1
What learning needs do you identify based on your previous clinical placements? How do you view the nursing process? What are your thoughts on the nursing process from earlier clinical placements? How have you worked with the nursing process previously? What are your expectations of the reflection card?
Interview 2
How are you using the reflection card in your daily work? What is your experience of using the reflection card? Has the reflection card had any impact on your development? In what way can the reflection card support your development of clinical competence? What obstacles do you see in using the reflection card?
Interview 3
Has there been any change in how you use the reflection card in your daily work? How? Has the reflection card’s role in your development changed over time? How? Has the reflection card contributed to your development of clinical competence? How? Do you see any obstacles with the reflection card? Can the reflection card support you in your future professional role? How?

### Data analysis

Longitudinal qualitative analysis lacks standardised methods and is dependent on the research questions [26]. This study followed Kneck and Audulv’s [32] pattern-oriented analysis (POLA), which identifies diverse patterns of individual change over time. In the first step of the analysis, the focus was on students’ development through use of the reflection card across three interviews. The next step was to look at descriptions and understandings of students’ shared patterns and different processes of progressing through time. A shared pattern is an interpreted pathway through time which is represented by a subsample [32].

Before the POLA analysis was conducted, each interview was structured using inductive qualitative content analysis [33, 34]. Interviews were read several times to grasp the context, and meaning units were identified and condensed based on the study aim. Thereafter, for each interview, the condensed meaning units with similar content were sorted into subcategories, which were subsequently merged and abstracted into broader categories (Table 2). The process was iterative, with continual comparison with the transcripts to ensure accuracy. The authors discussed and refined the analysis until consensus was reached. After further interpretation, a theme was developed by identifying the common thread running through the subcategories and categories in each interview. The analysis was conducted using NVivo 13 [35].

**Table 2:** An example of the analytical process applied to one student’s interview data (student 2)

Individual pattern	Enhances understanding of the nursing process as an integrated part of clinical practice	Develops independent reflective thinking in the professional role	Advances a fundamental structure for nursing practice	Continual reflective supervision
Theme	Be able to understand the application of the nursing process	Supports independent reflection on the patient	Structure in work	Provides structure for reflection with a supervisor
Category	Consolidates the nursing process	Using the card independently	Helps to structure the work	The supervisors have not used the card
	The nursing process becomes too abstract	Provides support in patient assessments	The card provides support in prioritisation Practical to bring along	Provides structure for reflection with supervisors
Theme	Support in applying the nursing process	A pedagogical tool for clinical reasoning	Support for documentation	Lack of reflection is a barrier
Category	Makes the nursing process more concrete	Contributes to reflection Using the card afterwards for reflection	Provides help with documentation	Reflection is deprioritised
Theme	Makes the nursing process a natural part of the work			
Category	Clarifies the nursing process			
	Helps maintain focus on nursing			
Theme	Support in preparation for assessment discussions			
Category	Provides preparatory support for assessment discussions			
	Helpful during the practical examination			

In the second stage, a summary was formulated based on the content of the subcategories, categories, and themes in order to facilitate the process of discovering individual patterns in each student, in line with Saldaña’s approach [26]. The analysis focused on identifying individual patterns in the changes and differences in students’ experiences regarding learning and development across various occasions. Individual patterns were then compared (Table 3), which revealed consistent main patterns across students (Table 4). Even when differences were minimal, attention to potential variation remained important. Matrices were used to organise and visualise the analysis process, as recommended by Saldaña [26].

**Table 3:** Example of the analysis of one main pattern across students

Main pattern: Enhances understanding of the nursing process as an integrated part of clinical practice						
	Interview 1		Interview 2		Interview 3	
	Student 1	Student 4	Student 1	Student 4	Student 1	Student 4
Theme	Be able to understand the application of the nursing process	Receive support in applying the nursing process	Support in applying the nursing process	Support in preparation for assessment discussions	Have the nursing process naturally made visible	Support throughout the entire education
Category	Understand what a structured nursing process is	Apply the nursing process together with a supervisor	Apply the nursing process	Tool for the assessment discussion	The card clarifies the nursing process in practice	Receive the card in earlier semesters
	The nursing process is difficult in practice	The nursing process occurs automatically				

### Ethical considerations

Confidentiality was ensured, and participation was voluntary, with the right to withdraw at any time. The researchers had no prior relationship with the students, and those who were interested initiated contact and provided their written consent.

## Results

Four main patterns (Table 4) were identified which illustrate students’ development of clinical competence over time through the use of reflection prompts during their clinical education. The clarity of these patterns’ appearance among the students varied and expressions of the patterns occurred at different points during the interviews, indicating that learning and development is not a linear process. The extent of students’ use of the card differed for various reasons, and the contribution to their learning and development differed accordingly.

Patterns I, II, and IV were present to varying degrees and from different perspectives among all students, while Pattern III was identified among four students. These four students described more in depth a transformative process linked to the use of the prompts, which provided them with a new understanding of the nursing process that they had not previously held during their education. They reported that the reflection prompts improved their reflective abilities and increased their awareness of the importance of reflection in professional practice. By contrast, two students stated that they had not used the card or felt the need to do so. These students more frequently described a lack of time and space for reflection and use of the prompts, which limited the depth of any transformative process. All students stated that the supervisor’s role in relation to the card was insufficient.

**Table 4:** Overview of main patterns

Main patterns
I. Enhances understanding of the nursing process as an integrated part of clinical practice
II. Develops independent reflective thinking in the professional role
III. Advances a fundamental structure for nursing practice
IV. Continual reflective supervision

### Enhances understanding of the nursing process as an integrated part of clinical practice

The nursing process was described by the students as abstract, difficult to apply in practice, and something that takes time to learn. They stated that it was not until later semesters that they began to understand what a structured nursing process entails. The process was perceived as complicated, as the steps are carried out but are not clearly visible in practice. It was considered to be two separate entities in theory and practice, wherein the process often occurs more automatically and unconsciously in practice.

*“It’s so good in theory*,* but when you’re actually on a ward*,* it feels like you do it automatically*,* but you don’t really think carefully about the different steps.”* (Student 3, interview 1).

The prompts provided support in reinforcing the application of the nursing process, as they helped to facilitate comprehensive understanding through reflection—both individual and with supervisors—on the different steps. The prompts served as a tool to translate the process into practice, concretising its various steps and helping make it less abstract. They were used to structure information and reflect on where in the process one was situated.

The prompts were also perceived as a support in clarifying what is done when one works based on the nursing process. In other words, the prompts facilitated an understanding of what a structured nursing process entails in practice. When the process became clear, it was perceived as a useful tool. To this end, the prompts contributed to raising awareness of the process and how to work based on it. Through repeated use, the understanding of the process’s structure increased, and thinking in line with the process became an established method integrated into the work.

*“The nursing process has been kind of more abstract before or like in the beginning. I’m starting to have these ’aha’ moments*,* it’s starting to click*,* or it’s starting to become more of a help for me*,* even in how I work.”* (Student 6, interview 2).

The concept of the nursing process had been present throughout the students’ education, but only now had it become more comprehensible, thanks to the contribution of the prompts. Students stated that the card would have been a valuable resource earlier during clinical education, to facilitate understanding and application of the nursing process. One student described that introducing the card earlier in the programme could have helped make the process more concrete in practice, which in turn could have supported their development in becoming a better nurse.

The prompts were also used as support in preparations for assessment discussions, by helping to structure examples of patient situations. It was also helpful in preparing and sitting for the practical examination, as it facilitated reflection based on the nursing process. However, one student shared that the card did not make a significant difference at this later stage of their education, as the work had become more independent and the focus had shifted more towards clinical practice rather than additional theoretical tools.

*“Honestly*,* I haven’t used it much at all*,* maybe mentioned it once. Towards the end*,* I’ve been so independent that I haven’t really needed it. It’s like I’ve found another way into it. Everything has gone well*,* and I just haven’t felt the need to use it.”* (Student 5, interview 3).

### Develops independent reflective thinking in the professional role

Students explained that the reflection prompts provided support and structure for reflecting on where in the process they were and what could have been done differently. Reflection with the prompts was experienced as more conscious and continuous, which might not have occurred otherwise. Students explained that the prompts had developed their ability to reflect by encouraging them to think things through one more time.

*“It makes you think twice*,* reflect a bit more*,* because I have the card. It makes you*,* well*,* take an extra moment to consider and reflect: can I change something to achieve a better outcome?”* (Student 4, interview 2).

Students described that the reflection prompts supported them in reflecting independently on their patient, especially when there was no time for reflection with a supervisor; it provided support in reflecting on the conversation with the patient both before and after data collection, to identify needs and risks during problem-solving and when evaluating interventions. The prompts developed their clinical competence by reminding them to reflect on aspects which might otherwise be forgotten. In this way, they supported the creation of a more structured and comprehensive view of the situation.

*“It’s based on those questions*,* but then you don’t really think as deeply when you’re just thinking in your head*,* kind of. That’s why it’s been helpful when I’ve then looked at the questions and go through a scenario afterwards*,* with the help of the card. That’s what gives you more answers about what you’ve done*,* like more understanding.”* (Student 4, interview 3).

Students’ reflections contributed to improvements in their next situation. Students also shared that reflections based on the prompts deepened their understanding of the patient and supported the development of clinical competence across various situations, because reflecting on the link between theory and practice deepened their understanding of what is done, how, and why. They felt that the prompts supported critical thinking in situations, which helped them in their role as nurses working in collaboration with other professions. Accordingly, they developed into more reflective and questioning professionals, which contributed to increased independence and confidence in being able to stand on their own and demonstrate their knowledge. The prompts also supported self-reflection on areas for improvement and what knowledge the student needed to bring into future patient situations.

*“I do something that I know is right because I’ve learned it visually*,* kind of like from memory*,* but I actually have no idea why. I don’t want to do something without knowing the reason*,* there should be a purpose behind it. So in that way*,* the card has helped me a lot. Really good.”* (Student 6, interview 3).

### Advances a fundamental structure for nursing practice

The card was perceived as a practical tool to carry and served as valuable support during patient encounters. It became a reminder in daily work, something students could return to for support and structure in clinical practice. The prompts contributed to creating structure in nursing care and facilitated students’ understanding and justification of their actions, which in turn helped them with prioritisation. The students explained that the prompts gave them a structure for independent work, which was applicable in all types of situations, by encouraging preparation and reflection in each patient situation. The prompts supported them during patient assessments and data collection, by helping to ensure that nothing was missing. It also facilitated the assessment and evaluation of a nursing diagnosis, as well as the follow-up of interventions. When students received new patients, the prompts served as a support in identifying needs and risks based on the patient’s condition. The prompts also provided support in documentation, as it helped link the nursing process to documentation and the development of care plans with goals and interventions.

*“If we have a patient who desaturates significantly*,* then when I’ve used the card*,* it has helped me with structuring things*,* especially when evaluating different interventions. So I do think I’ve remembered to use it well.”* (Student 4, interview 2).

*“When you’re on a cardiology ward*,* there have been quite a few respiratory-related nursing diagnoses. In a way*,* [it’s] obvious that it’s highly prioritised since it’s about breathing. So I’ve probably used it a bit more when trying to identify what needs exist and what risks the patients have*,* and so on.”* (Student 2, interview 2).

The greatest obstacle students mentioned was remembering to use the card, especially in the fast-paced daily workflow. However, once the card was integrated into clinical practice, it was perceived not as time-consuming but as a support for working more efficiently through increased structure and thoughtfulness. The students expressed that the prompts would continue to be a helpful tool even after graduation, in their role as a newly graduated nurse.

*“You’re supposed to remember to use it. Yeah*,* it’s actually pretty easy to forget and sometimes things can be a bit hectic*,* and then there’s not much reflection in the moment.”* (Student 3, interview 2).

### Continual reflective supervision

The opportunity for continual reflection was perceived by the students as a crucial condition for learning and for developing clinical competence. It was important to reflect continually throughout the day, as this helped ensure that experiences were not forgotten and that reflection was anchored in clinical practice. To this end, the reflection prompts served as a support for reflecting with supervisors.

*“It’s been helpful from a learning perspective*,* especially when reflecting with my supervisor*,* because then you can go through how you were thinking*,* what interventions you did and whether you felt there was something you could have done differently or keep in mind for next time. That’s how I’ve used it*,* actually*,* and I even gave one of the cards to one of my supervisors.”* (Student 2, interview 3).

Students described a lack of structured reflection and insufficient reflection in supervision. While the card was intended to support this process, in many cases supervisors had used it only to a limited extent or not at all. The insufficient reflection with supervisors meant that students did not receive feedback on their areas for development, which in turn hindered their progression in clinical competence. In supervision, lack of time was a barrier both to using the card and to reflection in general. One student shared that reflection with the supervisor could be reduced to a review of completed tasks. Supervisors often preferred that reflection took place at the end of the shift, as it could be perceived as disruptive to the workflow. In addition, students noted that the card could be particularly useful in the earlier stages of clinical education, when supervisors are more present and available. In the later phases of the programme, reflections occurred more sporadically, as students became more independent and took greater responsibilities.

*“There are lot of admissions*,* and some days are really hectic*,* you have to give a lot of IV antibiotics*,* medications*,* do rounds and all the daily tasks. Sometimes you don’t have time to do anything beyond the basics. I don’t reflect with my supervisor when there’s so much else going on.”* (Student 3, interview 2).

*“Everything just keeps rolling all the time. It’s almost like you don’t have time to think about the card at all.”* (Student 5, interview 2).

Despite these obstacles, the students saw potential in the card. They believed that if the card had been integrated into supervision, reflection based in the nursing process could have become clearer and more structured. The card could serve as a tool during reflection sessions with supervisors by promoting more in-depth questions, which in turn would promote the student’s development of clinical competence.

## Discussion

The aim of this study was to identify patterns in nursing students’ learning and development of clinical competence through the use of reflection prompts during clinical education. The results identified four main patterns depicting how students developed their clinical competence over time when supported by the reflection prompts as learning aid. The use of the card varied among students, and its impact on their learning differed accordingly. A deeper transformative process was elucidated in which students gained a new understanding of the nursing process, developed insight into reflection, and became more aware of the importance of reflection in professional practice. However, obstacles to the use of the card were also revealed—related to lack of time, opportunities for reflection, and supervision—which limited the potential for deeper learning.

With Pattern I, the results revealed that the reflection prompts enhanced understanding of the nursing process as an integrated part of clinical practice. This aligns with the results of the conceptual model developed and evaluated by Koldestam et al. [18, 19, 36, 37], which emphasises the interaction between intrapersonal and contextual factors in supervision and patient encounters. Their conceptual model contributes to a learning structure where care and learning can be integrated. In the present study, reflection prompts grounded in the nursing process appeared to support intrapersonal development, including a deeper understanding of and a clearer focus on nursing in concrete patient situations. This study result adds to the perspective of using a theoretical model which is central to nursing as the foundation for reflection, as this approach enables reflection on the entire process of patient care and supports students in maintaining focus and developing an in-depth understanding of what nursing entails, in line with the purpose of Koldestam et al. conceptual model [19]. Overall, this highlights the importance of meaningful learning in education, where theoretical knowledge is followed up and reflected on in clinical settings, thereby ensuring a clear connection between theory and practice. Integrating theory and practice can be challenging in education, which is reflected in students’ understanding in this study’s results. To this end, a didactic model can also support supervisors’ pedagogical competence and thereby increase students’ ability to develop independence in their professional role, which was also highlighted by Koldestam et al. [18].

With Pattern II, it was revealed that the reflection prompts facilitated the development of independent reflective thinking in the professional role. Developing reflective thinking is described as an important clinical competence to cultivate in preparation for entering the nursing profession and for continued professional development [15, 23]. According to Klaeson et al. [38], critical reflection at the end of education involves being receptive to change, taking a step back, and questioning one’s own understanding. The students in this study expressed that the reflection prompts helped them pause and reflect on the situation. Reflecting and reason critically strengthened their independence and increased their confidence in their own competence. Klaeson et al. [38] described this as part of the critical reflection at the end of nursing education, where the nurse, due to their competence, can advocate for the patient.

Jaastad et al. [17] showed in their review that the process of becoming a professional nurse is a continuing journey towards a deeper understanding of oneself and one’s way of being, which requires external support. In the present study, it was found that the reflection prompts provided a theoretical structure for the reflection process and increased students’ awareness of the reflection process. This aligns with Jaastad et al. [17], who concluded that theory facilitates students’ understanding of their actions, their purpose, and the consequences, and how students can reflect on themselves in relation to theory. The current findings suggest that structured reflection supports students’ learning goals and development, which the reflection prompts facilitate through a concrete, theory-based framework. In this context, the prompts can be considered as part of the didactic structure. According to Widarsson et al. [23], such structures should be developed through collaboration between higher education and clinical practice, to integrate theoretical and practical knowledge and thereby promote students’ development of clinical competence.

In Pattern III, the reflection prompts were described as providing a fundamental structure for nursing practice. They offered a sense of safety, a supportive framework on which students could rely on in their daily work. Johnsson et al. [6] described how a structural model during clinical education contributes to a sense of safety in students’ practice. Having something to lean on gives students self-confidence and increases their independence. This aligns with the findings of the present study, which indicate that when the card—structured around the nursing process—became an established and conscious model with which students engaged, they experienced improved efficiency in their work. Consequently, it can be assumed that the prompts based on the nursing process support the development of clinical competence, as they are perceived as a pedagogical tool which enhances students’ understanding of the nurse’s area of responsibility [6].

Pattern IV reveals the importance of continual reflection for the card to fulfil its full potential in supporting students’ development of clinical competence. Reflection and feedback go hand-in-hand in enabling student growth, and a structured, systematic approach to feedback and reflection contributes to a well-functioning supervisory team, which is a responsibility shared by the student and supervisor [21]. The reflection prompts can not only support self-reflection and help students identify gaps in their competence but also assist supervisors by providing a structured framework for reflection and feedback. Openness to self-reflection and a willingness to engage in professional development are central aspects of students’ critical reflection [38]. However, support in the reflection process from supervisors is also essential to ensuring optimal development of clinical competence. To this end, the results of this study indicate that reflection is not always a natural or integrated part of clinical education, a finding also encountered by Widarsson et al. [23]. A possible reason why the student feels that the reflection card is unnecessary is that they already consider themselves sufficiently independent in their daily work. However, this perception of independence can be referred to being able to carry out tasks on their own. If the reflection card had been used more consistently and clearly in the supervision process, the student would likely have developed a better understanding of why reflection is important for professional development. Taken together, this needs to be strengthened so that all students are given the opportunity to reflect continually and systematically during their clinical education. Otherwise, there is a risk of task-oriented focus [8], and deeper professional development may be lost [17]. Reflection prompts based on the nursing process are one way to potentially strengthen both the student’s and the supervisor’s role in learning and thereby contribute to the student’s development of clinical competence.

### Methodological discussion

To ensure transparency and quality, the study followed the COREQ checklist for qualitative research [39] and applied the trustworthiness criteria: credibility, dependability confirmability, and transferability [33].

Credibility in this study was ensured through several strategies. Although the study was short-term in duration, its design was guided by its aim, consistent with Saldaña’s [26] view that duration in QLS should be purpose-driven. A shorter longitudinal study enhances recall and relevance for students. In accordance with Audulv et al. [30], the data collection and analysis methods were carefully selected to match the research questions. The interviews provided rich data, and saturation was gradually reached [33], which supported the sample size. Additionally, there were no dropouts among the students, which is common in longitudinal studies [26]. As Saldaña [26] noted, limited data or a short study duration may hinder credible observations of change, which could occur after the study ends. Change is also often subtle and not explicitly expressed, and so interpretative analysis is required to uncover individual and shared patterns over time. In this study, while change was interpreted at a deeper level, the analytical process from condensation to categorisation was conducted at a manifest level of low abstraction. This study’s credibility is supported by the fact that all participating students met the nursing course objectives and achieved sufficient clinical competence to work as newly graduated nurses. What needs to be kept in mind is that the study was conducted within the context of a single educational setting, which is a limitation and may introduce bias.

Dependability was strengthened by ensuring the study’s consistency and transparency in the analytical process. Since data was collected over time, new questions may have emerged which were not covered during the initial data collection [26]. This study could have further developed questions regarding where the student was in their learning process and what kind of change they expected in their learning. Change occurs for many reasons and has interconnected effects [26]. Learning and the development of clinical competence is a complex process influenced by various factors such as prior experiences and individual characteristics, which makes it difficult to attribute changes solely to the intervention. In a clinical educational context with clear pedagogical organisation, structure and competence in supervision, the results might have looked different, and the students might have had a similar learning experience even without the reflection card. Additionally, students’ initial level of competence and the quality of supervision they receive are significant factors which influence their development. Supervision and the learning environment are situated in diverse settings, which further impacts students’ progress. A key strength of this small-scale study is the opportunity to refine data management and intervention procedures. Supervisors’ involvement is crucial for the further development of the intervention, as their active engagement can increase its implementation and effectiveness.

Confirmability can be discussed through the authors’ dual roles as nurses and educators, which provided valuable insight but also posed a risk of pre-understanding. Long-term engagement in the study may influence student, and researchers must remain neutral to strengthen confirmability. To strengthen confirmability, the researchers engaged in ongoing reflection and comparison with the original data.

While the study may be transferable to similar contexts, differences in educational and healthcare settings must be considered. All participants in this study came from the same nursing programme, which is a limitation and must be considered when transferring the results. In addition, future research should adopt a longitudinal design across the full three-year programme to track clinical competence development.

## Conclusion and implications

Reflection prompts based on the nursing process show the potential to support nursing students’ learning and development of clinical competence during clinical education. However, further research is needed to refine and strengthen the model, as well as to explore how it can be implemented in nursing education. Findings from this study highlight the importance of a well-structured approach throughout nursing education to ensure progression in learning in relation to the nursing process. Reflection prompts grounded in the nursing process can support both students and supervisors in bridging theoretical knowledge and practical application. By promoting the integration of theory and practice, the prompts can contribute to a deeper understanding of the nursing process among students and thereby promote meaningful learning throughout the educational journey. The prompts provided a structured framework for both reflection and clinical practice. As a didactic learning tool, the prompts have the potential to increase clinical competence by encouraging a more critically reflective approach, deepening understanding of patient situations, and reinforcing a conscious work structure that supports independence. To ensure that the reflection prompts provide optimal support for student learning, it is essential that clinical supervisors receive appropriate training and are actively involved in implementation of the prompts.

## Data Availability

The datasets generated and analysed during the current study are not publicly available due individual privacy, but are available from corresponding author on reasonable request.
